# Comparison of endotracheal aspirate and bronchoalveolar lavage fluid metagenomic next-generation sequencing in severe pneumonia: a nested, matched case–control study

**DOI:** 10.1186/s12879-023-08376-9

**Published:** 2023-06-12

**Authors:** Renren Bao, Qing Mei, Tianjun Yang, Lei Zhang, Chunyan Zhu, Xiaoqin Fan, Yinzhong Wang, Fei Tong, Yuxi He, Xiaowei Fang, Shike Geng, Yu Yang, Ximei Sheng, Aijun Pan

**Affiliations:** 1 Department of Critical Care Medicine, The Affiliated Provincial Hospital of Anhui Medical University, Hefei, 230001 Anhui China; 2 Department of Critical Care Medicine, Division of Life Sciences and Medicine, The First Affiliated Hospital of USTC, University of Science and Technology of China, Hefei, 230001 Anhui China; 3 WanNan Medical College, Wuhu, 241002 Anhui China

**Keywords:** Next-generation sequencing, Bronchoalveolar lavage fluid, Endotracheal aspirate, Severe pneumonia, Intensive care unit

## Abstract

**Objectives:**

To compare clinical outcomes in patients with severe pneumonia according to the diagnostic strategy used.

**Methods:**

In this retrospective, nested, case–control study, patients with severe pneumonia who had undergone endotracheal aspirate (ETA) metagenomic next-generation sequencing of (mNGS) testing (*n* = 53) were matched at a ratio of 1 to 2 (*n* = 106) by sex, age, underlying diseases, immune status, disease severity scores, and type of pneumonia with patients who had undergone bronchoalveolar lavage fluid (BALF) mNGS. The microbiological characteristics and patient’s prognosis of the two groups were compared.

**Results:**

An overall comparison between the two groups showed no significant differences in bacterial, fungal, viral, or mixed infections. However, subgroup analysis of 18 patients who received paired ETA and BALF mNGS showed a complete agreement rate for the two specimens of 33.3%. There were more cases for whom targeted treatment was initiated (36.79% vs. 22.64%; *P* = 0.043) and fewer cases who received no clinical benefit after mNGS (5.66% vs. 15.09%; *P* = 0.048) in the BALF group. The pneumonia improvement rate in the BALF group was significantly higher than in the ETA group (73.58% vs. 87.74%, *P* = 0.024). However, there were no significant differences in ICU mortality or 28-day mortality.

**Conclusions:**

We do not recommend using ETA mNGS as the first-choice method for analyzing airway pathogenic specimens from severe pneumonia patients.

**Supplementary Information:**

The online version contains supplementary material available at 10.1186/s12879-023-08376-9.

## Introduction

Severe pneumonia is a critical disease caused by pathogenic microorganisms’ invasion of lung tissue. If treatment is delayed, severe pneumonia can develop into respiratory failure or even multiple organ dysfunction. The occurrence and development of severe pneumonia are very rapid and fatal, and one study has reported that mortality is as high as 30%–50% [1]. In addition to necessary mechanical ventilation, early and effective anti-infective treatment is particularly important for patient prognosis. Obviously, the accurate and timely identification of pathogens can help clinicians choose the correct treatment regime [2].

Traditional methods for routine pathogen detection have many shortcomings. The positive rate of bacterial/fungal cultures is low and is affected by the degree of standardization of specimen collection and identification [3]. Additionally, cultures are time-consuming in many clinical laboratories, and a waiting time of 3–5 days is necessary for an accurate report. Serological tests and PCR are common virus detection methods; however, these require a priori hypothesis for the existence of specific viruses and laboratories to have corresponding commercial kits. These conditions usually lead to the missed detection of some pathogens. In recent years, the rapid development of metagenomic next-generation sequencing (mNGS) technology has provided new methods for pathogen diagnosis. The establishment of localized experimental platforms can even provide valid etiological information within 10–24 h. In addition, as an unbiased detection method, mNGS can simultaneously detect a diverse range of microorganisms, including bacteria, fungi, and viruses, in clinical specimens [4].

Other key factors that affect the efficacy of the identification of pathogenic microorganisms are the selection and collection of clinical specimens. Tissue or secretions obtained from the primary lesion are the ideal specimens. Most patients with severe pneumonia receive endotracheal intubation and mechanical ventilation. Thus, bronchoalveolar lavage fluid (BALF) is naturally regarded as a valuable specimen type. Our previous study showed that diagnosis using mNGS of BALF in ventilator associated pneumonia (VAP) is feasible and accurate, and mNGS has a higher sensitivity and specificity than traditional detection methods [5]. However, BALF acquisition requires fiberoptic bronchoscopy, which is relatively time-consuming and complex. Moreover, expensive fiberoptic bronchoscopy is a scarce resource in many intensive care units (ICUs), and it is often difficult to meet the needs of patients with respiratory diseases. Therefore, endotracheal aspirate (ETA) is often used as a substitute clinical specimen because it is convenient to obtain. Traditionally, ETA was considered inferior to BALF as an etiological specimen for lower respiratory tract infections; however, Kalantar et al. recently used mNGS to compare BALF and ETA and showed that their microbiomes are very similar [6]. This report piqued our curiosity over whether the characteristics of mNGS can improve the diagnostic value of ETA for lower respiratory tract infections. In this matched case–control study, we compared the microbiological diagnosis results obtained from mNGS of the ETA and BALF of patients with severe pneumonia and assessed the impact of analyzing the two approaches on patient outcomes.

## Methods

### Study population

This retrospective case-matched study was conducted in the First Affiliated Hospital of the University of Science and Technology of China, a tertiary grade-A hospital in China with a 130-bed ICU. After approval by the ethics committee of our hospital, an electronic database was established for all patients with pulmonary infection admitted to the ICU from March 2018 to February 2022. The following inclusion criteria were used: (i) met the diagnostic criteria for severe pneumonia according to 2007 Infectious Diseases Society of America (IDSA)/American Thoracic Society (ATS) guidelines [7]; and (ii) BALF or ETA specimens were collected and sent to the laboratory for mNGS within 24 h after the patient was admitted to the ICU. The following exclusion criteria were used: (i) presence of infection at sites other than the lungs; (ii) patients who died or were discharged automatically within 48 h of being admitted to the ICU; (iii) patients admitted to the ICU after being diagnosed with severe pneumonia for more than 48 h; (iv) patients considered to have had non-infectious disease in retrospective analysis; (v) ETA and BALF specimens did not meet mNGS quality control standards (e.g. library concentration < 50 pmol/L; Q20 < 85%; Q30 < 80%; guanine-cytosine content > 45%; total reads < 10 million); (vi) specimens were contaminated (e.g. that contaminant sequences are inversely correlated with total sequencing reads; that contaminant sequences are present in more controls than samples [8]); and (vii) clinical data were incomplete. Data were collected from the hospital’s electronic medical records, including demographic characteristics, underlying diseases, primary disease, disease severity scores, microbiological information, laboratory indicators, treatment course, and prognosis.

### Case matching

**Fig. 1:**
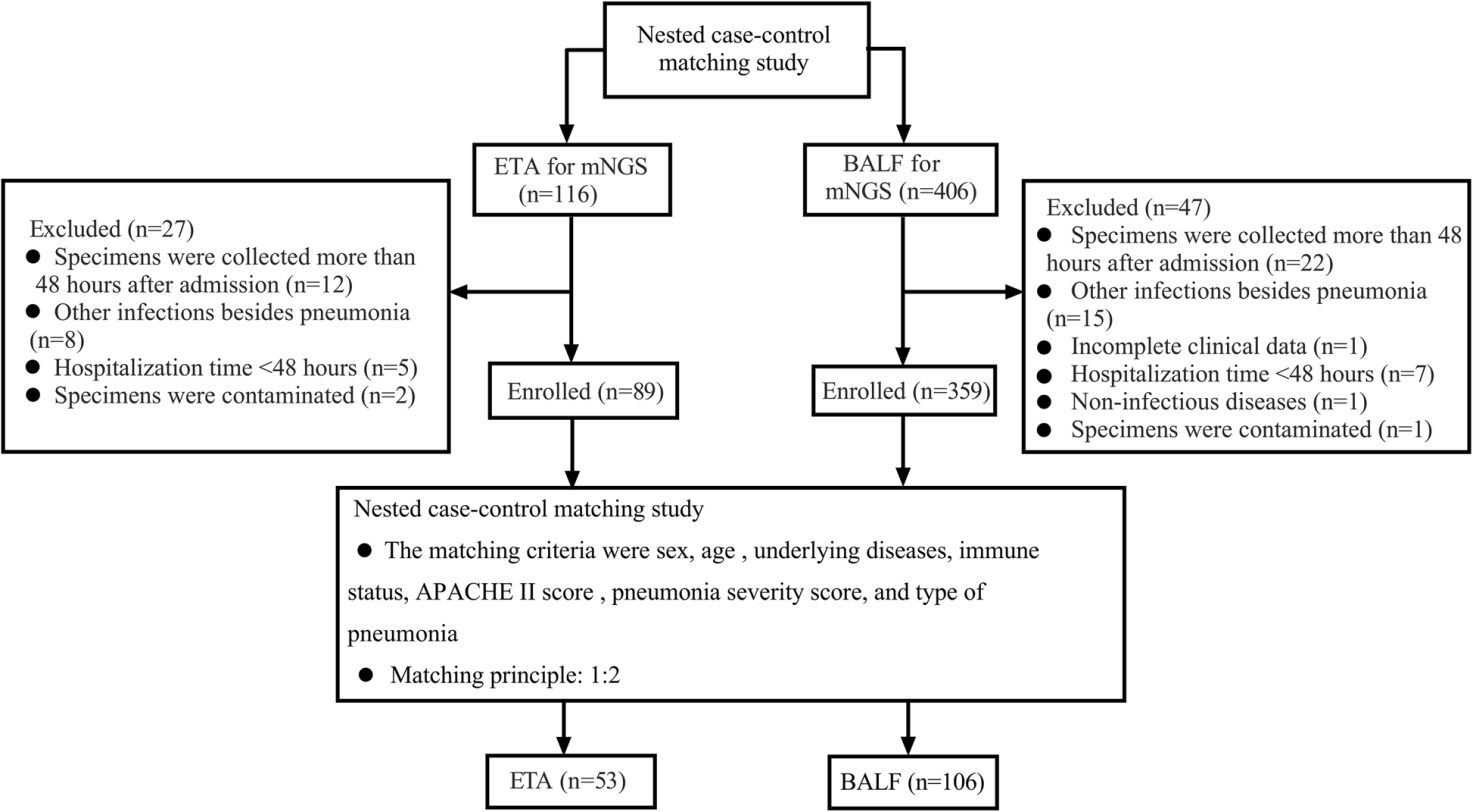
Flow diagram. Abbreviation: ETA endotracheal aspirates; BALF bronchoalveolar lavage fluid; APACHE II, acute physiology and chronic health evaluation II

The 1:2 pairing principle was adopted; that is, each patient with severe pneumonia who underwent ETA mNGS examination was matched with two patients who underwent BALF mNGS. The matching criteria were sex, age (within 5 years), underlying diseases, immune status, APACHE II score (within 3 points), pneumonia severity score (within 5 points on the same level), and type of pneumonia (hospital-acquired/community-acquired) (Fig. 1). When multiple control candidates met the core matching criteria, the choice was based on ICU admission dates. Investigators were blinded to case outcomes during matching. The primary endpoint was 28-day mortality, and the secondary endpoints were anti-infective regimen adjustment, improvement in pneumonia, ICU length of stay, total mechanical ventilation duration, and ICU mortality.

### Subgroup analysis

To understand whether there were differences between ETA mNGS and BALF mNGS in the microbial etiological diagnosis of the same patient, ETA specimens from 18 patients in the BALF group described above were collected on the day of BALF collection. The specimens were stored in a − 80 °C freezer, and mNGS was performed centrally.

### Conventional microbiological tests

ETA and BALF specimens were obtained as previously described [9, 10]. All specimens were divided into two after collection, with one of these specimens being delivered to a laboratory for further analysis within 1 h. The conventional microbiological tests (CMT) used in this study were showed in Additional file 1: Table S1. Culture and smear microscopy (except for special staining) were performed for each sample. Other CMT were conducted according to the necessity of clinical assessment. All patients after January 2020 received reverse transcription-polymerase chain reaction (PCR) testing for corona virus disease 2019. The protocols and primers of PCR for detecting 17 pathogens were shown in Additional file 2: Table S2.

### mNGS

The other specimen, described above, were sent to the Center for Accurate Diagnosis of Infectious Diseases established by USTC-BGI for mNGS. Two hundred and fifty μL of 0.5-mm glass beads was added to 600 μL of specimen for physical wall breaking; then 7.2 μL Lyticase (RT410-TA, Tiangen Biotech, Beijing, China) was added for an enzymatic wall breaking reaction. According to the steps of the TIANamp Micro DNA Kit (DP316, Tiangen Biotech), 300 μL of the mixture was used for DNA extraction. The extracted nucleic acids were enzymatically fragmented, end repaired, and ligated with adapters, and PCR were used to construct the library. All libraries were quantified by Qubit High Sensitivity DNA assay and distribution and quality assessed on a Bioanalyser DNA 1000 Chip on an Agilent 2100 (Agilent Technologies, USA) before normalizing and pooling. The qualified DNA library pool was circularized to form a single-stranded circular structure; and rolling circle amplification was used to generate DNA nanoballs. The prepared nanoballs were loaded on the chip, and the metagenomic sequencing platform BGISEQ-50/MGISEQ-2000 was used for sequencing.

After the sequencing was complete, linker sequences and low-quality reads were removed from the raw data to obtain high-quality clean data. The sequencing reads after quality control were mapped to human reference databases to remove human reads via Burrows-Wheeler-Alignment (BWA). After low-complexity reads were removed, the remaining sequence data were aligned to the current bacterial, viral, fungal, and protozoan databases, which were downloaded from the PMDB pathogen database (BGI) (https://bioinformatics.cineca.it/PMDB/). The database used for the present study contains 6,350 bacterial, 1,064 fungal, 4,945 viral, and 234 parasite species that are associated with human diseases.

### Criteria for positive mNGS results [5, 11]

- 1. A database of background microorganisms was established that contained microorganisms appearing in more than 50% of the specimens in the laboratory in the previous 3 months. Then, the detected microorganisms were compared with the in-house database to delete suspected background microorganisms.
- 2. To determine whether the microorganisms detected in specimens were truly positive, the reads per million (RPM) of the detected microorganisms were compared with those in a negative specimen.
- 3. Microorganisms with a high absolute abundance (≥ 30%) at the genus level were selected as potential pathogens.
- 4. For fungi, the result was considered positive if the species was detected by mNGS with an RPM ≥ 5.
- 5. For Mycobacteria and *Legionella pneumophila*, results were considered positive if mNGS detected species with ≥ 1 RPM.
- 6. The number of specific sequences for accurate identification of species should cover at least 3 different regions of the species genome.
- 7. Clinical factors were used to determine whether the microorganisms detected had clinical significance. Clinical factors included clinical manifestations, laboratory tests, imaging examinations, and treatment responses.

The above criteria were judged by a group of senior clinicians, who reached a unified opinion. The reliability of the pathogen diagnosis was divided into four levels.

- 1. Confirmed: the clinical manifestations were consistent with pathogen diagnosis; the specific treatment was effective; and there was good evidence from a diagnostic method other than mNGS, such as concordant results obtained by culture or smear.
- 2. Probable: although the diagnosis could not be fully confirmed, the clinical manifestations were consistent with pathogen diagnosis and the specific treatment was effective.
- 3. Possible: the diagnosis could not be fully confirmed; some clinical manifestations were consistent; and specific treatment was effective or ineffective.
- 4. Unlikely: the microorganisms belong to respiratory background microorganisms; these were contaminating microorganisms and theoretically pathogenic but completely inconsistent with clinical manifestations.

### Clinical assessment

The adjustment of anti-infective drugs and the outcomes of pneumonia were retrospectively judged by the same group of senior clinicians based on clinical data and the mNGS results. Anti-infective drug use in patients after receiving mNGS was divided into the following four categories: treatment confirmation, treatment de-escalation, initiation of targeted treatment, and no clinical benefit. The therapeutic effect on pneumonia after mNGS was judged according to symptoms, signs, and laboratory test results. If the following two indicators were met, the treatment was defined as effective: (1) pneumonia symptoms improved or disappeared (sputum properties improved; pulmonary rales decreased or disappeared; peak body temperature decreased; and peripheral blood leukocyte, neutrophil ratio, C-reactive protein, procalcitonin, and other inflammatory indicators tended to be normal); and (2) chest imaging suggested that the lesions were absorbed or there was no apparent progression.

### Statistical analysis

Measurement data conforming to normal distribution were expressed as mean ± standard deviation (SD), and t-test was used for comparison between groups. The normally distributed measurement data were expressed as median (interquartile range), and comparisons of specimens between two groups were performed using nonparametric tests. Categorical variables were expressed as numbers (percentages), and chi-square tests were used for comparisons between two groups. Survival analysis was performed with Cox regression model and Kaplan–Meier survival curve analysis. SPSS 23.0 software was used for statistical analysis of data, and a *P* value of ≤ 0.05 was considered to be statistically significant.

## Results

A total of 1787 patients were diagnosed with severe pneumonia in four ICU centers. Among them, 522 patients with severe pneumonia underwent ETA or BALF mNGS within 24 h of ICU admission. Finally, 53 patients who underwent ETA were successfully matched with 106 patients who underwent BALF (Fig. 1). Table 1 shows the characteristics of the patients who had an ETA mNGS test and their respective controls. No significant differences in demographics, disease severity, primary disease, or laboratory examinations were observed between the two groups. In addition, no significant differences were found between the two groups in terms of the time from onset to ICU admission, time from ICU admission to specimen collection, or inflammatory indicators.

**Table 1:** Demographic and clinical characteristics of the patients in the ETA group and BALF group

	ETA Group (*n* = 53)	BALF Group (*n* = 106)	*P* value
Age (years), mean ± sd	66.12 ± 15.98	65.42 ± 16.49	0.150
Gender (female), n (%)	14 (26)	28 (26)	1
Type of pneumonia (HAP), n (%)	41 (87.4)	82 (87.4)	1
APACHE II score, mean ± sd	16.30 ± 3.36	16.90 + 2.98	0.372
**PSI, n (%)**			
Grade III	13 (24.5)	26 (24.5)	1
Grade IV	39 (73.6)	78 (73.6)	1
Grade V	1 (1.9)	2 (1.9)	1
**Primary disease, n (%)**			
Disease of cardiovascular system	25 (47.2)	50 (47.2)	1
Autoimmune diseases	12 (22.6)	24 (22.6)	1
Diabetes	12 (22.6)	24 (22.6)	1
Diseases of urinary system	11 (20.8)	22 (20.8)	1
Nervous system diseases	8 (15.1)	16 (15.1)	1
Disease of respiratory system	3 (5.7)	6 (5.7)	1
Tumor	5 (9.4)	10 (9.4)	1
Others	3 (5.7)	6 (5.7)	1
None	3 (5.7)	6 (5.7)	1
Time from diagnosis of severe pneumonia to ICU admission (h), median (IQR)	20 (10.25, 35)	22.5 (13.25, 35.75)	0.499
Time from ICU admission to specimen submission (h), median (IQR)	9.5 (4, 13)	14 (5, 19)	0.400
Time from specimen submission to the intervention (h)	22.5 (19.5, 26)	23 (19, 26.5)	0.379
**Laboratory examination**^a^			
White blood cell count (× 10^9^/L), mean ± sd	12.47 ± 6.31	11.94 ± 6.71	0.709
Neutrophils (%), mean ± sd	86.38 ± 7.27	87.80 ± 7.87	0.420
CRP (mg/L), median (IQR)	87.83 (49.83, 180.86)	85.64 (60.12, 117.37)	0.899
PCT (ng/mL), median (IQR)	1.31 (0.208, 7.62)	1.223 (0.314, 6.2)	0.785

*Abbreviation*: *ETA* Endotracheal aspirates, *BALF* Bronchoalveolar lavage fluid, *sd* standard deviation, *HAP* Hospital acquired pneumonia, *APACHE II* Acute physiology and chronic health evaluation II, *PSI* Pneumonia severity index, *CRP* C-reactive protein, *IQR* Interquartile range, *PCT* Procalcitonin, *ICU* Intensive care unit

^a^Laboratory test results of the day of mNGS specimen collection

The comparison of bacterial, fungal and viral detection by the CMT and mNGS methods is shown in Additional file 3: Figure S1. The percentage of mNGS-positive samples was significantly higher than that of CMT-positive samples with regard to bacterial and fungal detection (*P* < 0.001). Although this difference is significantly reduced at the species level, only a few pathogens were detected by CMT only no matter in ETA group or BALF group. Additional file 4: Figure S2 shows the concordance analysis between CMT and mNGS. In the ETA group, a total of 14 (26.4%) cases were positive by mNGS only, whereas 2 (3.8%) cases were positive by CMT only. Of 32 patients who were positive by both methods, the results of 8 (15.1%) patients were completely matched and the results of 4 (7.5%) patients were completely mismatched. The remaining 20 (37.7%) patients were “partially matched”, indicating that at least one pathogen detected by the two methods overlapped. Similar results were observed in BALF group. A total of 25 (23.6%) cases were positive by mNGS only, whereas 3 (2.8%) cases were positive by CMT only. Of 70 patients who were positive by both methods, the results of 18 (17.0%) patients were completely matched and the results of 7 (6.6%) patients were completely mismatched. The remaining 45 (42.5%) patients were partially matched.

**Fig. 2:**
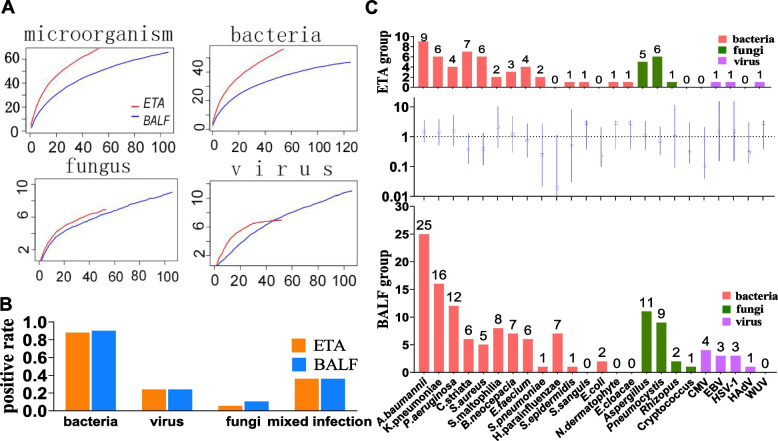
Different microbiome in the ETA group and BALF group. **A** Comparison of species accumulation curves for the total microorganism, bacteria, fungus and virus between the ETA group (red lines) and BALF group (blue lines). **B** Distribution of different types of microbial etiology. **C** Spectra of potential pathogens in the ETA group and BALF group. Blue error line (across the grid lines) in the middle panels indicate that pathogens were not significantly different between the two groups (*P* > 0.05). Abbreviations: ETA, endotracheal aspirates; BALF, bronchoalveolar lavage fluid; CMV, Cytomegalovirus; EBV, Epstein-Barr virus; HSV-1, Herpes simplex virus type 1; HAdV, Human adenovirus; WUV, WU polyoma virus

Figure 2A shows species accumulation curves for total microorganisms, bacteria, fungi, and viruses detected in 53 ETA and 106 BALF specimens. No significant differences were found between the two groups in terms of fungi and viruses detected, while the total numbers of microbial species and bacterial species detected by mNGS in ETA specimens were significantly higher than those detected in BALF (*P* < 0.001). However, when only potential pathogenic microorganisms were considered, there was no statistical difference between the ETA and the BALF detection of bacterial, fungal, and viral infections (*P* > 0.05) (Fig. 2B). Figure 2C shows the differences in the genus or species level identification of potential pathogens between the two groups. There were 127 and 268 potential pathogenic microorganisms identified in the ETA group and BALF group, respectively. Common bacteria such as *Acinetobacter baumannii, Klebsiella pneumoniae, Pseudomonas aeruginosa,* and *Staphylococcus aureus* remained the most frequently detected pathogens in the two groups. In addition, *Corynebacterium striata*, which is not normally reported by traditional detection methods, was frequently detected both in the BALF and ETA groups. Seven cases of *Haemophilus parainfluenzae* were detected by mNGS from BALF, but none from ETA. The most frequently detected fungi in the two groups were *Aspergillus* and *Pneumocystis*. All instances of cytomegalovirus (*n* = 4) detection were in the BALF group. However, there was no statistically significant difference between the two groups at each genus or species level.

**Fig. 3:**
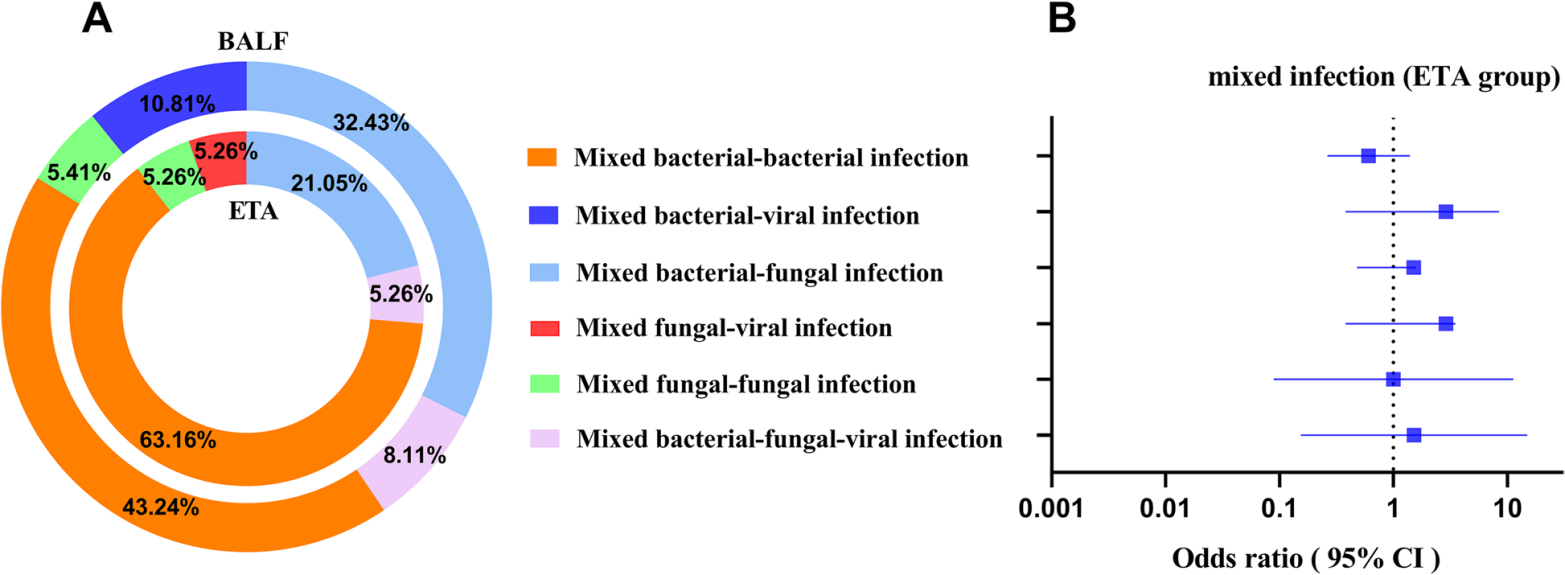
Mixed-infection in the ETA group and BALF group. **A** Constitutional ratios of mixed-infection in 53 patients undergoing ETA mNGS (inner circle) and 106 patients undergoing BALF mNGS (outer circle). **B** Mixed-infection in the ETA group. The square symbols represent odds ratio, and the horizontal lines represent the 95% confidence interval. Abbreviations: ETA, endotracheal aspirates; BALF, bronchoalveolar lavage fluid

Infection with more than one pathogen is termed a mixed infection. The mNGS analysis of ETA and BALF diagnosed 19 (35.8%) and 38 (35.8%) mixed infections, respectively (*P* = 1.0). The most common types in both groups were mixed bacterial–bacterial infections, followed by mixed bacterial–fungal infections. There were no statistically significant differences between the two groups, even when specific mixed infection types were considered (Fig. 3).

We performed a subgroup analysis of the 18 patients who received paired ETA and BALF mNGS. Additional file 5: Table S3 presents the potential pathogens identified by mNGS of both specimens. The complete agreement rate of the two specimens was 33.3%, the partial agreement rate was 55.6%, and the nonconformity rate was 11.1%.

Although clinicians use mNGS to identify some microorganisms as potential pathogens, there is a different level of reliability for each pathogen (Additional file 6: Table S4). Table 2 shows a comparison of the reliability of the diagnosis of potential pathogens between the two groups on a level scale, where unlikely pathogens were not shown. The BALF group had more confirmed pathogens and less possible pathogens than the ETA group. Therefore, BALF had obvious advantages over ETA for pathogenic microorganism diagnostic reliability.

**Table 2:** The reliability of the microbial etiological diagnosis in the ETA and BALF groups

	ETA (*n* = 53)	BALF (*n* = 106)	*P* value
Confirmed	12	42	0.029
Probable	23	55	
Possible	30	38	

*Abbreviation*: *ETA* Endotracheal aspirates, *BALF* Bronchoalveolar lavage fluid

**Fig. 4:**
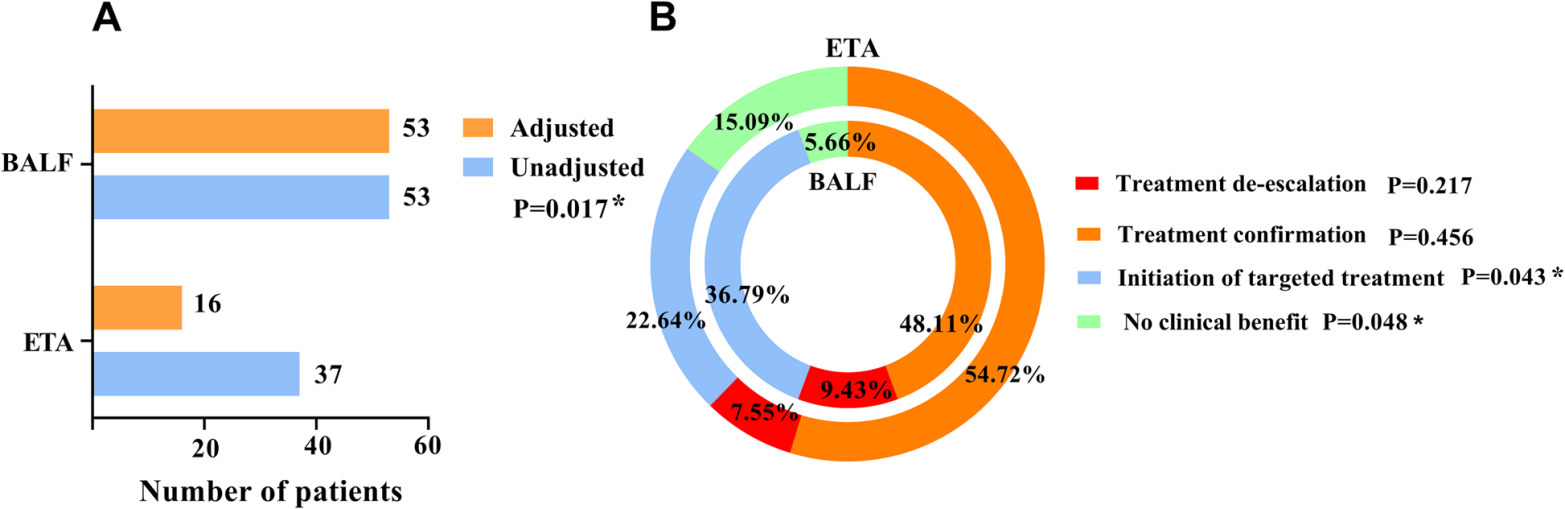
Impact on anti-infective regimens. **A** Adjustment of anti-infective regimens after mNGS. **B** Four specific subcategories of anti-infective regimens after mNGS in ETA groups (outer circle) and BALF groups (inner circle). Abbreviations: ETA endotracheal aspirates; BALF bronchoalveolar lavage fluid; mNGS, metagenomic next-generation sequencing

Clinicians adjusted the anti-infective regimen of each patient based on the microbiological reports. We observed that the proportion of anti-infective regimen adjustments for BALF mNGS was significantly higher than that for ETA mNGS (50% vs. 30.19%; *P* = 0.017) (Fig. 4A). The initiation of targeted treatment in the BALF group was significantly higher than that in the ETA group (36.79% vs. 22.64%; *P* = 0.043), whereas no difference in treatment de-escalation was observed between the two groups. For no adjustment, there were more cases of no clinical benefit from mNGS in the ETA group than in the BALF group (15.09% vs. 5.66%; *P* = 0.048), whereas there was no significant difference in treatment confirmation between the two groups (Fig. 4B).

**Fig. 5:**
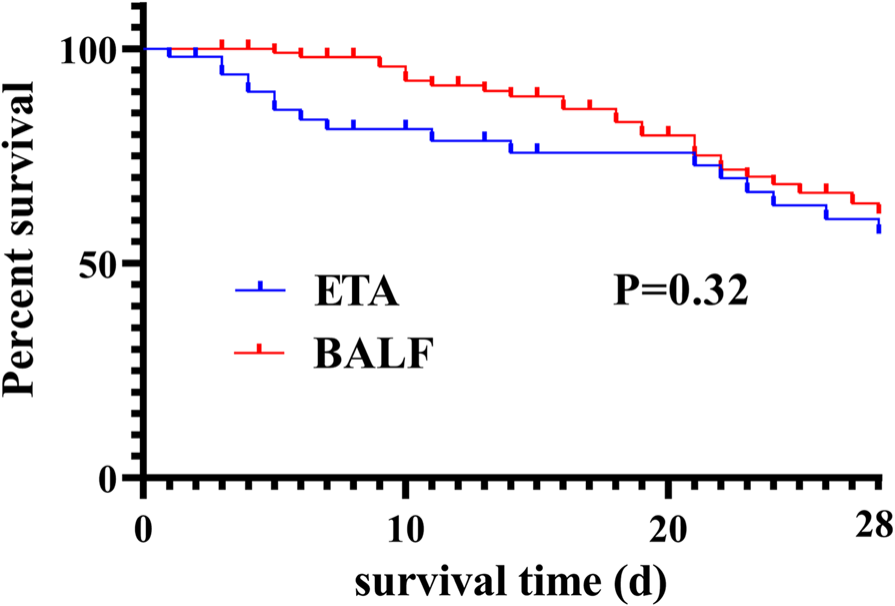
Kaplan–Meier curves for 28-day mortality in the ETA groups (blue lines) and BALF groups (red lines). *P* = 0.32, OR = 1.373%; CI = 0.7342–2.568. Abbreviations: ETA, endotracheal aspirates; BALF, bronchoalveolar lavage fluid

Table 3 shows the prognosis of the two groups of patients. The improvement rate of pneumonia in the BALF group was significantly higher than that in the ETA group (*P* = 0.024). In addition, the duration of mechanical ventilation (*P* = 0.031) and the duration of ICU stay (*P* = 0.049) were also shorter in the BALF group than the ETA group. However, there were no significant differences in ICU mortality or 28-day mortality, which was further confirmed by the Kaplan–Meier survival analysis. The 28-day survival rates of the ETA and BALF groups were 56.92% and 61.51%, respectively (*P* = 0.32), i.e., not statistically significant (Fig. 5).

**Table 3:** Comparison of the prognosis between the two groups

	ETA (*n* = 53)	BALF (*n* = 106)	*P* value
Improvement of pneumonia, n (%)	39 (73.58)	93 (87.74)	0.024
Mechanical ventilation time (d), median (IQR)	11.00 (1.25, 21.25)	9 (4, 18.25)	0.031
Length of ICU stay (d), median (IQR)	22 (11, 36)	14 (10.25, 26.75)	0.049
Mortality in ICU, n (%)	14 (26.4)	22 (20.8)	0.271
28-day mortality, n (%)	18 (33.96)	28 (26.42)	0.32

*Abbreviation*: *ETA* Endotracheal aspirates, *BALF* Bronchoalveolar lavage fluid, *IQR* Interquartile range, *ICU* Intensive care unit

## Discussion

Similar to other research [5, 12, 13], our results indicated that the mNGS was superior to the CMT methods for detecting common bacteria and fungi. However, the value of ETA compared with BALF as an etiological diagnostic specimen of the lower respiratory tract infection is controversial. Most studies have demonstrated that ETA is more sensitive and easily influenced by commensal bacteria in the oral cavity and upper respiratory tract, resulting in a failure to detect pathogenic microorganisms when traditional methods are used. Ranzani et al. analyzed 200 patients with hospital-acquired pneumonia who were assessed using invasive and non-invasive approaches for microbiological diagnosis. The results showed that BALF had higher rates of successful microbiological diagnosis compared with sputum and ETA [14]. More recently, however, several studies have suggested that ETA has a tendency to surpass BALF when molecular diagnostic methods are used [6, 15] Indeed, with the development of mNGS technology, it is now possible to assess airway microbiota without relying on culture-based methods. This revolutionary technique brings unprecedented resolution and provides an opportunity to evaluate the differences between ETA and BALF [6].

In this study, we compared the microbiological mNGS results for 53 ETA and 106 BALF specimens. Species accumulation curves suggested that more types of microorganisms were present in the ETA specimens, which was mainly attributed to their higher bacterial diversity. However, this difference may have been caused by commensal microorganisms, because when only potential respiratory pathogens were considered, there were no significant differences in bacteria, fungi, or viruses between the two groups. Even when comparing at the genus or species level, the pathogen detection rates did not differ between the two groups. Moreover, BALF did not show obvious advantages in terms of judging the various types of mixed infections. The respiratory pathogen profiles obtained by ETA mNGS and BALF mNGS appear to be similar in patients with severe pneumonia. This finding is consistent with the results of a prospective cohort study conducted by Kalantar et al. [6]. However, the above findings were obtained through an overall comparison between two groups, and they did not provide information on the consistency of the diagnostic results for the two groups. For the individual patient, the situation may be completely different. We performed subgroup analysis in which both ETA and BALF were obtained from the same individual to identify pathogenic microorganisms using mNGS, and the results showed that there were many partial concordances, or even discordances between the two methods, which may lead to differences in treatment decisions.

To directly focus on the real pathogen among the multitude of microorganisms reported in the mNGS report is challenging and requires careful consideration. Currently, no unified standard for diagnosis is available, and physicians rely on a comprehensive analysis of clinical symptoms, laboratory test results, and imaging data. In this study, clinicians made subjective judgments about the potential pathogens in each mNGS report, but they had a significantly lower confidence in the diagnosis results for the ETA group than those for the BALF group. This lack of confidence was mainly based on the lower degrees of accordance between the pathogenic microorganisms identified and the clinical characteristics of the patients. In addition, the interference of contaminating microorganisms colonizing the mouth and airways often affects the judgment of clinicians.

We noticed that patients in the ETA group received a lower proportion of anti-infective regimen adjustments. The incidence of initial targeted therapy in the ETA group was significantly lower than that in the BALF group, whereas more patients received no clinical benefit in the ETA group than in the BALF group. This suggests that the patients in the ETA group received inferior anti-infective regimens compared with the BALF group. We cannot make causal claims linking inferior anti-infective regimens with the lower confidence of clinicians in the diagnosis for the ETA group, but the fact is that inferior anti-infective regimens ultimately resulted in a lower improvement of pneumonia in the ETA group. This also demonstrates that clinicians’ concerns about the diagnostic reliability of pathogenic microorganisms in the ETA group were justified.

The differences in the duration of mechanical ventilation and ICU length of stay also corroborated the evidence that patients in the BALF group received better anti-infective treatment. However, there were no differences in ICU mortality or 28-day mortality between the two groups, and patients in the BALF group did not appear to ultimately benefit from treatment. This may be because hospital acquired pneumonia (HAP) accounted for the majority (87.4%) in this study. The HAP patients were often associated with severe primary disease, and the prognosis of patients was affected not only by the effectiveness of the therapeutics for pneumonia but also by the treatment of the primary disease.

The present study contains certain limitations. The patients enrolled had only undergone DNA sequencing, whereas RNA sequencing was not performed simultaneously. Although only one RNA virus was detected in 46 samples by PCR, this means that the omission of RNA viruses does exist in our study. In addition, HAP accounted for 87.4% in this study. The significance of mNGS on HAP and community acquired pneumonia may be different [16, 17]. However, due to the low sample size, we did not conduct a subgroup analysis accordance with different types of pneumonia to clarify the impact of different sample types on pathogenic diagnosis and patient prognosis.

## Conclusion

Our study showed that some differences in the microbiological diagnosis via ETA mNGS and BALF mNGS appear to exist. Clinicians also expressed a lack of confidence in the diagnostic results of ETA. Although BALF mNGS did not result in better long-term prognosis compared with ETA mNGS, we still do not recommend ETA mNGS as the first-choice method for diagnosing airway pathogenic specimens from severe pneumonia patients, given the greater improvement in pneumonia, shorter mechanical ventilation time, and shorter ICU stay in the BALF group.

## Supplementary Information

**Additional file 1:** **Table S1.** Conventional microbiological tests used in this study.

**Additional file 2: Table S2.** The protocols and primers of real-time quantitative PCR for detecting 17 pathogens.

**Additional file 3: Figure S1.** The overlap of positivity between mNGS technique and CMT for different pathogens. The pathogens were observed to have a higher positive rate by mNGS than that by CMT, and the difference was significant. Abbreviation: ETA, endotracheal aspirates; BALF, bronchoalveolar lavage fluid; mNGS, metagenomic next-generation sequencing; CMT, conventional microbiological tests.

**Additional file 4: Figure S2.** Concordance analysis between mNGS and CMT method. For the double-positive subset, the results of the two methods were divided into completely matched, partial matched, and completely mismatched. Abbreviation: ETA, endotracheal aspirates; BALF, bronchoalveolar lavage fluid; mNGS, metagenomic next-generation sequencing; CMT, conventional microbiological tests.

**Additional file 5:** **Table S3.** Potential pathogens identified by paired ETA and BALF mNGS.

**Additional file 6:** **Table S4.** The reliability of potential pathogens in each patient.

## Data Availability

The datasets generated and/or analysed during the current study are available in the National Center Biotechnology Information BioProject database under accession number PRJNA846267.

## Abbreviations

mNGS: Metagenomic next-generation sequencing
ETA: Endotracheal aspirates
BALF: Bronchoalveolar lavage fluid
VAP: Ventilator associated pneumonia
CMT: Conventional microbiological tests
PCR: Polymerase chain reaction
RPM: Reads per million
HAP: Hospital acquired pneumonia
APACHE II: Acute physiology and chronic health evaluation II
PSI: Pneumonia severity index
CRP: C-reactive protein
SD: Standard deviation
IQR: Interquartile range
PCT: Procalcitonin
ICU: Intensive care unit
CMV: Cytomegalovirus
EBV: Epstein-Barr virus
HSV-1: Herpes simplex virus type 1
HAdV: Human adenovirus
WUV: WU polyoma virus
