# Supplementary material for: Comparison of endotracheal aspirate and bronchoalveolar lavage fluid metagenomic next-generation sequencing in severe pneumonia: a nested, matched case–control study
Source: BMC Infect Dis. 2023 Jun 12;23:389. doi: 10.1186/s12879-023-08376-9 (PMC10258078; doi:10.1186/s12879-023-08376-9)
Supplement: Supplementary file 1 — Additional file 1: Table S1. Conventional microbiological tests used in this study. [file 12879_2023_8376_MOESM1_ESM.pdf]

Table S1. Conventional microbiological tests used in this study

Conventional Microbiological Test	
(1)	Bacterial culture and smear microscopy (n=159)
(2)	*Mycobacterium tuberculosis* or NTM:<br>Acid-fast staining (n=17)<br>GeneXpert MTB/RIF (n=3)
(3)	PCR test for *Legionella, Mycoplasma pneumoniae* and *Chlamydia pneumoniae* (n=19)
(4)	Fungal culture and smear microscopy (n=159)
(5)	BALF galactomannan test (n=73)
(6)	BALF or sputum Grocott's methenamine silver stain (n=13)
(7)	PCR test for 8 RNA virus: influenza A H7N9, influenza A H1N1, influenza A H3N2, influenza B virus, parainfluenza virus, metapneumovirus, respiratory syncytial virus, rhinovirus (n=46)
(8)	PCR test for SARS-CoV-2 (n=42)
(9)	PCR test for adenovirus, boca virus, CMV、EBV and HSV (n=57)

Abbreviations: NTM, nontuberculous mycobacteria; MTB, Mycobacterium tuberculosis; RIF, rifampicin; PCR, polymerase chain reaction; BALF, bronchoalveolar lavage fluid; RNA, Ribonucleic Acid; CMV, cytomegalovirus; EBV, Epstein-Barr virus; HSV, herpes simplex virus; SARS-CoV-2, severe acute respiratory syndrome coronavirus 2.
