# Supplementary material for: Comparison of endotracheal aspirate and bronchoalveolar lavage fluid metagenomic next-generation sequencing in severe pneumonia: a nested, matched case–control study
Source: BMC Infect Dis. 2023 Jun 12;23:389. doi: 10.1186/s12879-023-08376-9 (PMC10258078; doi:10.1186/s12879-023-08376-9)
Supplement: Supplementary file 2 — Additional file 2: Table S2. The protocols and primers of real-time quantitative PCR for detecting 17 pathogens. [file 12879_2023_8376_MOESM2_ESM.pdf]

Table S2. The protocols and primers of real-time quantitative PCR for detecting 17 pathogens

Pathogen	Primer and probe	Temperature (℃)			The number of cycles
		Reverse transcription	denaturation	annealing	
Legionella	F 5'-TCCGGAAGCAATGGCTAAA-3'<br>R 5'-AGAACGTCTTTCATTTGCTGTTC-3'<br>P 5'-FAM-AGACGCTATGAGTGGCGCTCAA TT-TAMRA-3'	48	95	60	40
Mycoplasma pneumoniae	F 5'-GCAAGGGTTCGTTATTTG-3'<br>R 5'-CGCCTGCGCTTGCTTTAC-3'<br>P 5'-AGGTAATGGCTAGAGTTTGACTG-3'	45	93	55	40
Chlamydia pneumoniae	F 5'-AGCACAAACACCTCAGACTACAC-3'<br>R 5'-AGAACAATGCCGATI'CCTAAG-3'<br>P 5'-FAM-ACAACCATCAGTATCTCACAAGGCA ACAC-BHQ1-3'	48	95	60	40
Influenza A H7N9	H7-F 5'-AAAATAGAATACAGATWRACCCR GT-3'<br>H7-R 5'-GTGCACYGCATGTTTCC-3'<br>H7-P 5'-FAM-CTTCGGGGCATCATGTTTYMTW CTTCTRG-TAMRA-3'<br>N9-F 5'-CCAAATCAGAAGATTCTATGCACYT-3'<br>N9-R 5'-GGTTTGCYATTCCWATGARYA-3'<br>N9-P 5'-HEX-GCCACTGCYATYRTAATA-MGB-3'	42	94	60	40
Influenza A H1N1	F 5'-AAATCTAGTGGTACCGAGATATGCA-3'<br>R 5'-GGGAGGCTGGTGTTTATAGCAC-3'<br>P 5'-CAATGGAAAGAAATGCTGG-3'	50	95	55	40
Influenza A H3N2	H3-F 5'-AGGTGTTGAGCTAAATCTGGRTA CA-3'<br>H3-R 5'-GAACCCAGCAAAACAACACA-3'<br>H3-P 5'-FAM-TGGATTTCCTTTGCCATATCATG CTTTTTG-ECLIPSE-3'<br>N2-F 5'-TGGGAACCAAACAAGTGTGC-3'<br>N2-R 5'-CCTGTCATCCCCAGTGATACAA-3'<br>N2-P 5'-ROX-TAGCATGGTCCAGCTCAAGCTG CCAT-ECLIPSE -3'	45	94	60	40
Influenza B virus	F 5'-AGACCAGAGGGAAACTATGCCC-3'<br>R 5'-TCCGGATGTAACAGGTCTGACTT-3'<br>P 5'-FAM-CAGACCAAAATGCACGGGGAAHA TACC-BHQ1-3'	50	95	60	40
Parainfluenza virus	F 5'-GACCAGAAAATTATTGAAT -3'<br>R 5'-TACCAGGCACATGTGGGGTT -3'<br>P 5'-FAM-TACTGAGTCGGGCCAAGTA GC-MGB -3'	45	95	55	40

Metapneumo virus	F 5'-CAAGTGTGACATTGCTGAYCT RAA-3'<br>R 5'-ACTGCCGCACAACATTTAGRAA-3'<br>P 5'-TGGCYGTYAGCTTCAGTCAATTCAACA GA-3'	50	95	60	40
Respiratory syncytial virus	RSV-A-F 5'-ACTGCAATCAYACAAGATGCAA CRA-3'<br>RSV-A-R 5'-CAGATTGRAGAAGCTGATTCCA-3'<br>RSV-A-P 5'-FAM-CCAGATCAAGAACACAACC CCARCATACCT-BHQ1-3'<br>RSV-B-F 5'-ACTTACCTTACTCAAGTCTCACC AGAAA-3'<br>RSV-B-R 5'-TTGTRGCTGARTTTGTGTGGAT-3'<br>RSV-B-P 5'-Texas red-TTAGCCCATCCMAACA AYCCACAACC-BHQ2-3'	48	94	55	40
Rhinovirus	F 5'-TCCTCCGGCCCCTGAAT-3'<br>R 5'-GAAACACGGACACCCAAAGTAGT-3'<br>P 5'-TGGCTAACCCAAACCC-3'	42	95	60	40
SARS-CoV-2	Target 1(ORF1ab)<br>F 5'-CCCTGTGGGTTTTACACTTAA-3'<br>R 5'-ACGATTGTGCATCAGCTGA-3'<br>P 5'-FAM-CCGTCTGCGGTATGTGGAAAGGTTA TGG-BHQ1-3'<br>Target 2(N)<br>F 5'-GGGGAACTTCTCCTGCTAGAAT-3'<br>R 5'-CAGACATTTTGCTCTCAAGCTG-3'<br>P 5'-FAM-TTGCTGCTGCTTGACAGATT-TAM RA-3'	50	95	55	40
Adenovirus	ADV-BE-F 5'-GCACATCGCCGGACAGGA-3'<br>ADV-BE-R 5'-TTGTTCCCCAGAYTGAAGTAG GT-3'<br>ADV-BE- P 5'-ACTGCACCAGACCCGGACTSA GGTAC-3'<br>ADV-C-F 5'-GCCAGGACGCCTCGGAGTA-3'<br>ADV-C-R 5'-GTGGGGTTTCTAAACTTGTTATTC AG-3'<br>ADV-C-P 5'-CCGTGCCACCGAGACGTACTTCA GC-3'	45	95	60	45
Boca virus	F 5'-AGGAAGAGACACTGGCAGACAA-3'<br>R 5'-CAAGACGATAGGTGGCTGATTGG-3'<br>P 5'-FAM-TCATCACAGGAGCAGGAGCCGCAG C-TRAMA-3'	50	95	63	40
CMV	F 5'-GCTAACTATGCAGAG-CATGTA-3'<br>R 5'-GCTGAGTrcTGGTAAAGAAC-3'<br>P 5'-CTACAITGTACCCGAGGALFAAGCG-3'	48	93	55	40

EBV	F 5'-GTAGAAGGCCATTTTTCCAC-3'<br>R 5'-TTTCTACGTGACTCCTAGCC-3'<br>P 5'-FAM-ACCACCGTGGCCCAGATGG-TAMRA-3'	48	93	55	40
HSV	HSV-1-F 5'-ATACCGACCACACCGACGA-3'<br>HSV-1-R 5'-ACAACTCCCTAACCCCTGCT-3'<br>HSV-1-P 5'-TET-AGGGGCCATTTTACGAGGAGGA-BHQ-3'<br>HSV-2-F 5'-TTCCCCCGTGGCTCAATATT-3'<br>HSV-2-R 5'-ACGCGCCGGGGCAGGTCT-3'<br>HSV-2-P 5'-ROX-TTATGCCTATCCCCGGTTGGACGA-BHQ-3'	50	95	55	40

Abbreviations: PCR, polymerase chain reaction; SARS-CoV-2, severe acute respiratory syndrome coronavirus 2; CMV, cytomegalovirus; EBV, Epstein-Barr virus; HSV, herpes simplex virus.
