# Supplementary material for: Comparison of endotracheal aspirate and bronchoalveolar lavage fluid metagenomic next-generation sequencing in severe pneumonia: a nested, matched case–control study
Source: BMC Infect Dis. 2023 Jun 12;23:389. doi: 10.1186/s12879-023-08376-9 (PMC10258078; doi:10.1186/s12879-023-08376-9)
Supplement: Supplementary file 3 — Additional file 3: Figure S1. The overlap of positivity between mNGS technique and CMT for different pathogens. The pathogens were observed to have a higher positive rate by mNGS than that by CMT, and the difference was significant. Abbreviation: ETA, endotracheal aspirates; BALF, bronchoalveolar lavage fluid; mNGS, metagenomic next-generation sequencing; CMT, conventional microbiological tests. [file 12879_2023_8376_MOESM3_ESM.pdf]

Figure S1.

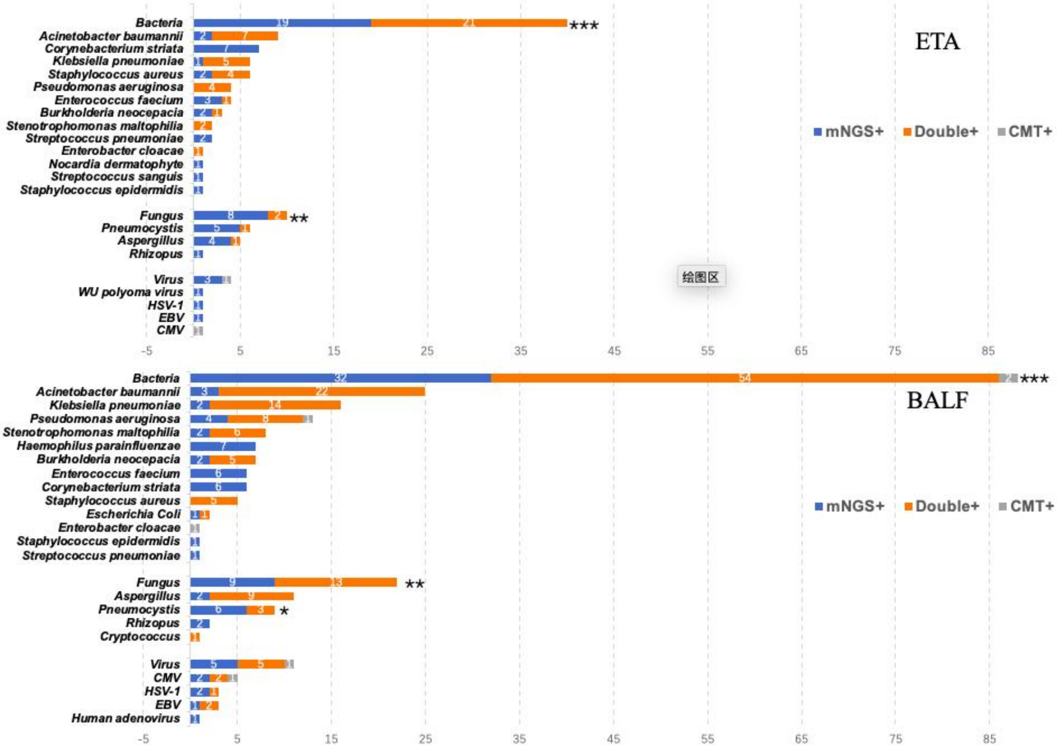

The overlap of positivity between mNGS technique and CMT for different pathogens. The pathogens were observed to have a higher positive rate by mNGS than that by CMT, and the difference was significant (* P<0.05, ** P<0.01, *** P<0.001).

Abbreviation: ETA, endotracheal aspirates; BALF, bronchoalveolar lavage fluid; mNGS, metagenomic next-generation sequencing; CMT, conventional microbiological tests.
