# Supplementary material for: Comparison of endotracheal aspirate and bronchoalveolar lavage fluid metagenomic next-generation sequencing in severe pneumonia: a nested, matched case–control study
Source: BMC Infect Dis. 2023 Jun 12;23:389. doi: 10.1186/s12879-023-08376-9 (PMC10258078; doi:10.1186/s12879-023-08376-9)
Supplement: Supplementary file 4 — Additional file 4: Figure S2. Concordance analysis between mNGS and CMT method. For the double-positive subset, the results of the two methods were divided into completely matched, partial matched, and completely mismatched. Abbreviation: ETA, endotracheal aspirates; BALF, bronchoalveolar lavage fluid; mNGS, metagenomic next-generation sequencing; CMT, conventional microbiological tests. [file 12879_2023_8376_MOESM4_ESM.pdf]

Figure S2.

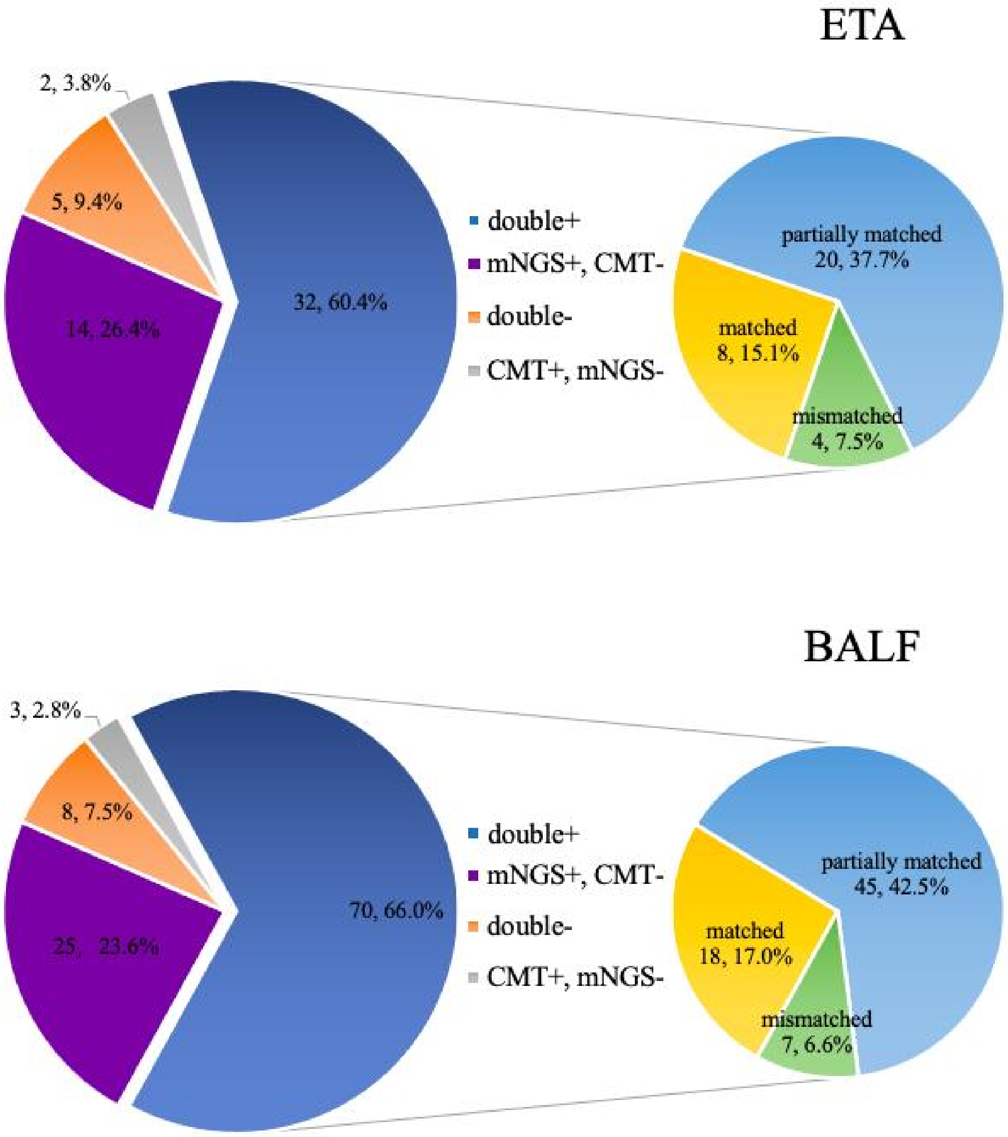

Concordance analysis between mNGS and CMT method. For the double-positive subset, the results of the two methods were divided into completely matched, partial matched (at least one pathogen detected by the two methods overlapped), and completely mismatched.

Abbreviation: ETA, endotracheal aspirates; BALF, bronchoalveolar lavage fluid; mNGS, metagenomic next-generation sequencing; CMT, conventional microbiological tests.
