# Supplementary material for: Comparison of endotracheal aspirate and bronchoalveolar lavage fluid metagenomic next-generation sequencing in severe pneumonia: a nested, matched case–control study
Source: BMC Infect Dis. 2023 Jun 12;23:389. doi: 10.1186/s12879-023-08376-9 (PMC10258078; doi:10.1186/s12879-023-08376-9)
Supplement: Supplementary file 5 — Additional file 5: Table S3. Potential pathogens identified by paired ETA and BALF mNGS. [file 12879_2023_8376_MOESM5_ESM.pdf]

Table S3. Potential pathogens identified by paired ETA and BALF mNGS.

ID	ETA			BALF			consistent
	bacteria	Fungi	Virus	bacteria	Fungi	Virus	
10	*S. pneumoniae*			*S. pneumoniae*			complete
12	*A. baumannii*<br>*S. aureus*			*A. baumannii*			partial
18	*A. baumannii*			*A. baumannii*			complete
25	*P. aeruginosa*<br>*K. pneumoniae*			*K. pneumoniae*			partial
30	*C. striata*		CMV	*A. baumannii*		CMV	partial
34	*A. baumannii*			*A. baumannii*			complete
39	*B. neocepacia*<br>*A. baumannii*			*B. neocepacia*	*Pneumocystis*		partial
40	*P. aeruginosa*			*P. aeruginosa*			complete
42	*C. striata*		EBV	*C. striata*		EBV	complete
44	*E. faecium*			*E.faecium*	*Aspergillus*<br>*Pneumocystis*		partial
45	*None*			*H. parainfluenzae*			inconsistent
47	*K. pneumoniae*<br>*C. striata*			*K. pneumoniae*			partial
50	*A. baumannii*			*S. maltophilia*			inconsistent
79	*A. Baumannii*<br>*B. neocepacia*			*K. pneumoniae*<br>*A. baumannii*			partial
91	*P. aeruginosa*	*Aspergillus*		*P. aeruginosa*			partial
105	*C. striata*			*C. striata*	*Aspergillus*		partial
111	*P. aeruginosa*	*Pneumocystis*			*Pneumocystis*		partial
154	*C. psittaci*			*C. psittaci*			complete

Abbreviations: ETA, endotracheal aspirates; BALF, bronchoalveolar lavage fluid; CMV, Cytomegalovirus; EBV, Epstein-Barr virus.
