# Supplementary material for: Comparison of endotracheal aspirate and bronchoalveolar lavage fluid metagenomic next-generation sequencing in severe pneumonia: a nested, matched case–control study
Source: BMC Infect Dis. 2023 Jun 12;23:389. doi: 10.1186/s12879-023-08376-9 (PMC10258078; doi:10.1186/s12879-023-08376-9)
Supplement: Supplementary file 6 — Additional file 6: Table S4. The reliability of potential pathogens in each patient. [file 12879_2023_8376_MOESM6_ESM.docx]

Table S4. The reliability of potential pathogens in each patient

|  | ETA (n=53) | | BALF (n= 106) | | | |
| --- | --- | --- | --- | --- | --- | --- |
|  | Confirmed | Probable | Possible | Confirmed | Probable | Possible |
|  | (n) | (n) | (n) | (n) | (n) | (n) |
| **Bacteria** |  |  |  |  |  |  |
| *Acinetobacter* *baumannii* | 0 | 1 | 5 | 0 | 4 | 1 |
| *Klebsiella* *pneumoniae* | 1 | 3 | 3 | 4 | 1 | 1 |
| *Corynebacterium* *striata* | 0 | 0 | 9 | 0 | 5 | 20 |
| *Staphylococcus* *aureus* | 0 | 1 | 1 | 0 | 1 | 0 |
| *Pseudomonas* *aeruginosa* | 0 | 1 | 0 | 0 | 0 | 0 |
| *Stenotrophomonas* *maltophilia* | 1 | 2 | 1 | 2 | 8 | 2 |
| *Burkholderia* *neocepacia* | 0 | 2 | 1 | 2 | 5 | 1 |
| *Enterococcusfaecium* | 1 | 3 | 2 | 3 | 10 | 3 |
| *Streptococcus* *pneumoniae* | 1 | 1 | 1 | 6 | 1 | 0 |
| *Haemophilus* *parainfluenzae* | 1 | 0 | 0 | 1 | 0 | 0 |
| *Staphylococcus* *epidermidis* | 0 | 2 | 2 | 2 | 3 | 1 |
| *Streptococcus* *sanguis* | 0 | 0 | 0 | 1 | 4 | 2 |
| *Escherichia* *Coli* | 0 | 0 | 0 | 1 | 1 | 0 |
| *Nocardia* *dermatophyte* | 1 | 0 | 0 | 0 | 0 | 0 |
| *Enterobacter* *cloacae* | 0 | 1 | 0 | 0 | 0 | 0 |
| *Chlamydia* *psittaci*  **Fungi** | 1 | 0 | 0 | 2 | 0 | 0 |
| *Aspergillus* | 2 | 2 | 1 | 7 | 3 | 1 |
| *Pneumocystis* | 1 | 0 | 0 | 2 | 0 | 0 |
| *Rhizopus* | 2 | 2 | 2 | 7 | 2 | 0 |
| *Saccharomyces* | 0 | 0 | 1 | 0 | 0 | 3 |
| *Cryptococcus*  **Virus** | 0 | 0 | 0 | 1 | 0 | 0 |
| CMV | 0 | 0 | 0 | 1 | 3 | 0 |
| EBV | 0 | 1 | 0 | 0 | 2 | 1 |
| HSV- 1 | 0 | 0 | 1 | 0 | 2 | 1 |
| HAdV | 0 | 0 | 0 | 0 | 0 | 1 |
| WUV | 0 | 1 | 0 | 0 | 0 | 0 |

Abbreviations: ETA, endotracheal aspirates; BALF, bronchoalveolar lavage fluid; CMV, Cytomegalovirus; EBV, Epstein-Barr virus; HSV- 1, Herpes simplex virus type 1; HAdV, Human adenovirus; WUV, WU polyoma virus.
